# The Distribution of *Toxoplasma gondii* Cysts in the Brain of a Mouse with Latent Toxoplasmosis: Implications for the Behavioral Manipulation Hypothesis

**DOI:** 10.1371/journal.pone.0028925

**Published:** 2011-12-14

**Authors:** Miroslava Berenreiterová, Jaroslav Flegr, Aleš A. Kuběna, Pavel Němec

**Affiliations:** Biology Section, Faculty of Science, Charles University in Prague, Praha, Czech Republic; Technion-Israel Institute of Technology Haifa 32000 Israel., Israel

## Abstract

**Background:**

The highly prevalent parasite *Toxoplasma gondii* reportedly manipulates rodent behavior to enhance the likelihood of transmission to its definitive cat host. The proximate mechanisms underlying this adaptive manipulation remain largely unclear, though a growing body of evidence suggests that the parasite-entrained dysregulation of dopamine metabolism plays a central role. Paradoxically, the distribution of the parasite in the brain has received only scant attention.

**Methodology/Principal Findings:**

The distributions of *T. gondii* cysts and histopathological lesions in the brains of CD1 mice with latent toxoplasmosis were analyzed using standard histological techniques. Mice were infected per orally with 10 tissue cysts of the avirulent HIF strain of *T. gondii* at six months of age and examined 18 weeks later. The cysts were distributed throughout the brain and selective tropism of the parasite toward a particular functional system was not observed. Importantly, the cysts were not preferentially associated with the dopaminergic system and absent from the hypothalamic defensive system. The striking interindividual differences in the total parasite load and cyst distribution indicate a probabilistic nature of brain infestation. Still, some brain regions were consistently more infected than others. These included the olfactory bulb, the entorhinal, somatosensory, motor and orbital, frontal association and visual cortices, and, importantly, the hippocampus and the amygdala. By contrast, a consistently low incidence of tissue cysts was recorded in the cerebellum, the pontine nuclei, the caudate putamen and virtually all compact masses of myelinated axons. Numerous perivascular and leptomeningeal infiltrations of inflammatory cells were observed, but they were not associated with intracellular cysts.

**Conclusion/Significance:**

The observed pattern of *T. gondii* distribution stems from uneven brain colonization during acute infection and explains numerous behavioral abnormalities observed in the chronically infected rodents. Thus, the parasite can effectively change behavioral phenotype of infected hosts despite the absence of well targeted tropism.

## Introduction

*Toxoplasma gondii* is a highly prevalent, intracellular protozoan parasite with an indirect life cycle. It infects a very broad spectrum of warm-blooded vertebrates, including humans, as intermediate hosts but can reproduce sexually only in the feline intestine [1]. The transmission of *T. gondii* is facilitated by its ability to modify the behavior of its intermediate hosts. Indeed, behavioral studies have gathered abundant evidence that latent toxoplasmosis is associated with impaired motor performance, deficits in spatial learning and memory, reduced anxiety, higher activity levels in both novel and familiar environments, sensory attention deficits, altered novelty seeking behavior, longer reaction times, and, most importantly, reduced avoidance of feline predators (for review, see [2]–[5]). These parasite-induced behavioral changes undoubtedly increase the predation risk in infected rodents and thereby enhance the likelihood of the parasite transmission to its definitive cat host. Moreover, the fact that infected rodents lose their innate fear of cat odor and develop a mild attraction to it, but retain the aversion to odors of other predators, which do not serve as definitive hosts of the parasite [6]–[9], cannot be explained as a by-product of parasitosis and clearly implies highly specific manipulation of the host behavior.

After acute infection characterized by fast asexual reproduction, *T. gondii* encysts in the brain (and other tissues) and remains and slowly asexually reproduces there for the host's lifetime. The parasite is thus well placed to affect the behavior of the infected individual. However, the proximate mechanisms by which *T. gondii* alters brain function remain largely unclear. The convergence of evidence, albeit at times circumstantial, suggests that synergic effects of three different mechanisms may underpin the *T. gondii* impact on local neural processing. First, chronic infection may result in a local degenerative cell loss. Parasites within neurons could directly cause the death of infected neurons or atrophy of their processes and inflammation may contribute, via the production of nitric oxide and other toxic oxygen products, to the death of neighboring neurons [10]. Because neurons greatly outnumber the *T. gondii* brain cysts, this phenomenon has been considered rather marginal until recently (e.g., [5]). However, recent magnetic resonance imaging studies reporting dilated ventricles in chronically infected mice [10] and reduced grey matter volume in *T. gondii* positive schizophrenia patients compared to that of *T. gondii* negative patients [11] are consistent with a significant loss of the brain parenchyma. Second, local immune responses required to keep *T. gondii* dormant may, through the production of proinflammatory cytokines, interferon-γ and indoleamine 2,3-dioxigenase, alter the levels, turnover and efficiency of many neuromodulators, including dopamine, glutamate and serotonin (for detailed discussion see [12], [13]). Finally, *T. gondii* may directly influence neurotransmitter levels. Whilst the association between increased dopamine levels and latent toxoplasmosis comes from the mid-eighties [14], recent studies have provided evidence strongly suggesting that the parasite directly increases local dopamine metabolism [15], [16]. More specifically, *T. gondii* contains genes encoding a tyrosine hydroxylase, a rate-liming enzyme of dopamine biosynthesis [15], and the encysted parasite expresses dopamine in vivo and enhances the K+-induced release of dopamine from dopaminergic neurons in vitro [16]. The implication of dopamine is supported by the hyperactivity of infected rodents [17]–[21], altered novelty seeking behavior in infected rodents [17], [18], [22], [23] and humans [12], [24,], and by the fact that dopamine agonists and antagonists disrupt the parasite-induced behavioral phenotypes [20], [25]. These findings reinforce the putative link between toxoplasmosis and schizophrenia (e.g., [26], [27]).

Paradoxically, the distribution of the parasite in the brain has received only scant attention (mouse: [21], [28]–[30]; immunocompromised mouse: [31]; rat: [7], [32]). Useful information also comes from neuroradiological and neuropathological examinations of human patients with AIDS (e.g., [33]–[36]). Most of the studies mentioned provide only a rough description of the parasite distribution and are based on arbitrarily chosen or equidistant sections. In this study, we therefore systematically mapped the distribution of *T. gondii* tissue cysts and histopathological lesions in the brains of mice with latent toxoplasmosis. Special attention was paid to the dopaminergic system and brain regions implicated in the modulation of defensive and aversive behaviors, spatial orientation and learning, and sensorimotor performance. The revealed distribution pattern elucidates mechanisms underlying parasite-induced behavioral changes.

## Results

### Clinical appearance

**Figure 1:**
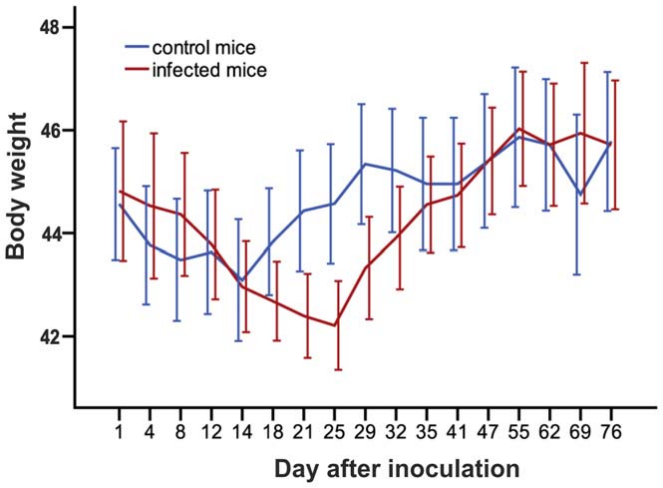
Transient reduction of body weight in the infected mice. The abscissa shows body weight, the ordinate shows time (in days) after inoculation. Error bars denote the 95% confidence interval.

Typical symptoms of acute toxoplasmosis, i.e., lethargy, ruffled fur or hunched posture were not apparent after peroral infection with 10 tissue cysts of the avirulent HIF *Toxoplasma* strain. Nevertheless, we observed a significant transient reduction of body weight in the infected mice from 21^st^ to 29^th^ day p.i. (GLM repeated measures, p = 0.017; Fig. 1). After this period, which likely coincided with the acute phase of toxoplasmosis, the body weights were no longer different between the infected and control mice (p = 0.783).

### Number and size of *T. gondii* cysts

**Figure 2:**
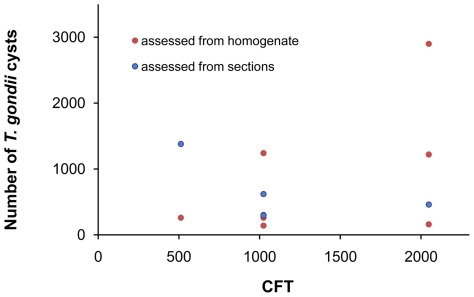
Numbers of brain tissue cysts and *T. gondii* antibody titers are not correlated. The abscissa shows total numbers of brain cysts, the ordinate shows antibody titers determined by the complement fixation test.

**Figure 3:**
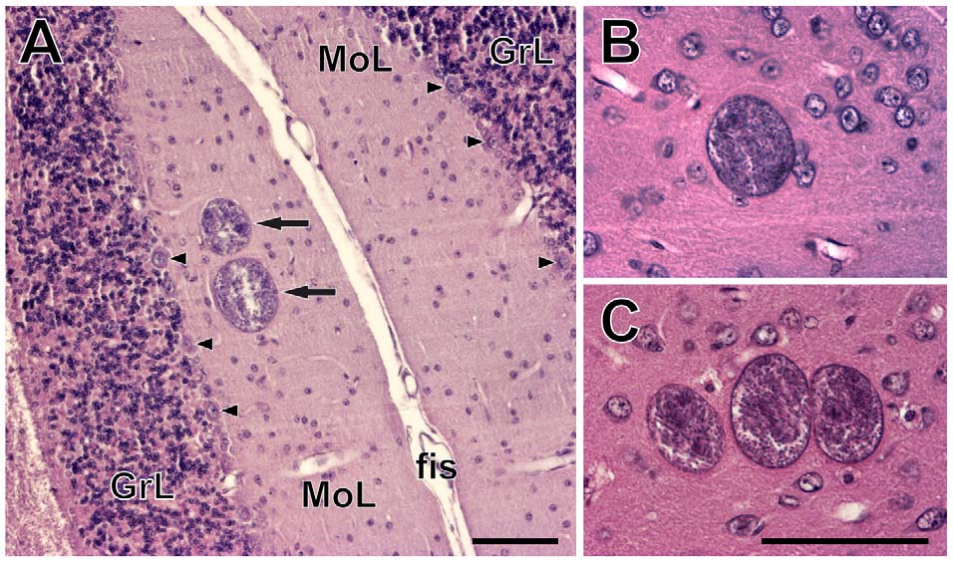
*Toxoplasma gondii* cysts in the brains of chronically infected CD1 mice. (A) A pair of cysts (arrows) in the molecular layer of the cerebellar cortex. Arrowheads point to the Purkinje cells. GrL, granular layer; fis, cerebellar fissure; MoL, molecular layer. (B–C) A single cyst (B) and a cyst triad (C) in the isocortex. Scales, 100 µm (in C for B, C).

Tissue cysts of *T. gondii* were found in brains of all infected mice. In the histologically processed brains, the numbers of brain cysts observed ranged between 297 and 1380 (mean ± s. d. = 611±402). The admittedly less reliable estimates based on brain homogenates showed a wider range between 140 and 2900 (mean ± s. d. = 883±938). It is unclear whether these latter counts reflect inter-individual differences in cyst density or technical limitations of the method. There was no relation between the anti- *T. gondii* titer and the number of brain tissue cysts (Kendall's τ = 0.093, p = 0.705; Fig. 2). The size of the cysts ranged between 10 and 70 µm. They were found solitary or in groups of 2–10 in hematoxylin-eosin stained sections (Fig. 3). On average, about 80% of the cysts were solitary, ∼13% of the cysts were found in pairs, ∼4% in triads; larger groups were rare.

Neither *T. gondii* cysts in the brain nor anti- *T. gondii* antibodies in the blood were detected in the control mice.

### Distribution of *T. gondii* cysts in the CNS

**Figure 4:**
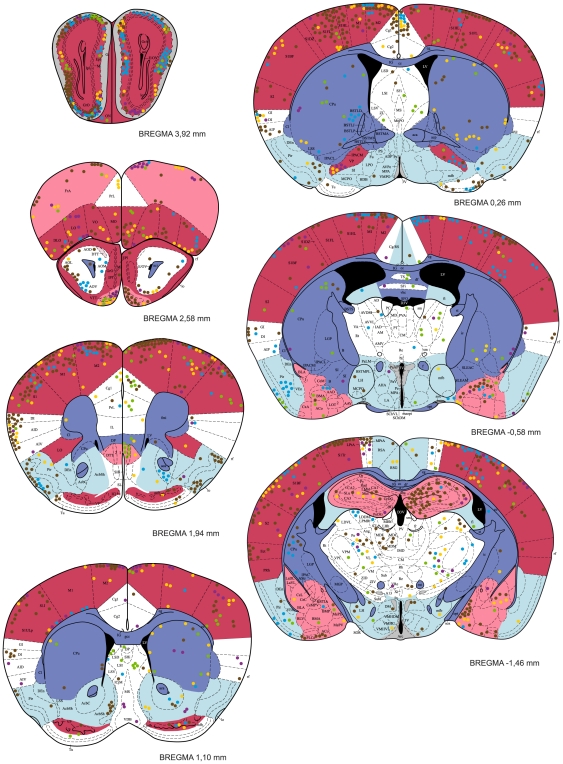
The distribution of *T. gondii* cysts in the forebrain of a mouse with latent toxoplasmosis. Coronal diagrams compile results from five infected CD1 mice. Tissue cyst locations are indicated by color circles (each circle represents a single cyst); colors refer to the individual animals investigated. The cyst density within brain regions is classified into five classes – very high, high, medium, low, and very low, which are colored red, pink, white, blue, and dark blue, respectively. Grey indicates the dopaminergic system. The antero-posterior positions of the sections are determined by Bregma coordinates. See Abbreviations S1 for abbreviations.

**Figure 5:**
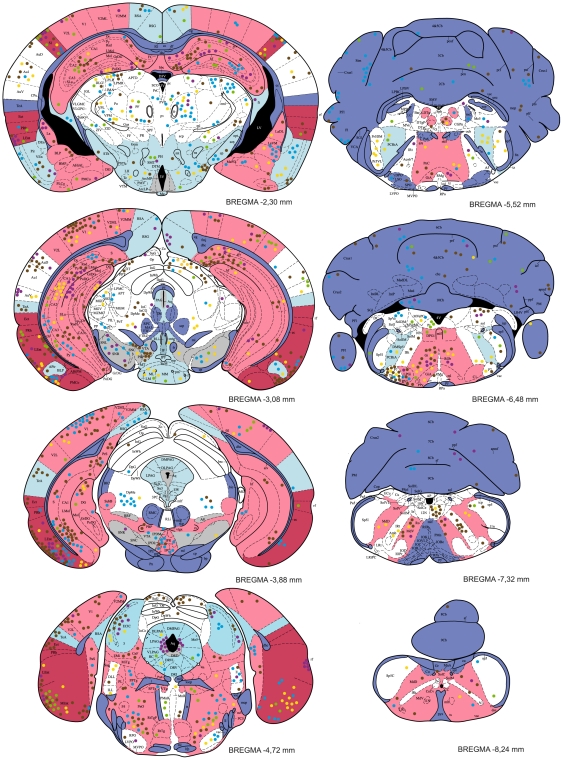
The distribution of *T. gondii* cysts in the caudal forebrain, the cerebellum and the brainstem of a mouse with latent toxoplasmosis. See caption to Fig. 4 for explanation.

Tissue cysts of the parasite were found in 54 anatomically defined brain regions, which altogether occupied 92% of the brain (Table 1). Locations of all cysts recorded within five brains examined in detail are shown in Figures 4, 5. The cysts were bilaterally distributed; no discernible lateralization was observed. However, the cyst density was not homogeneous, i.e., some brain regions were infected significantly more than others (GLMM, χ^2^ = 1086.4, p<10^−6^) and, importantly, brain region typical infestation levels featured a significant concordance among the infected mice (Kendall's W = 0.419, p<0.001). Among five fundamental brain parts, the telencephalon exhibited the highest cyst density. Comprising ∼56% of the mouse brain volume it contained ∼75% of cysts. Indeed, with very few exceptions, all consistently highly infected brain regions were located within the telencephalon (see below). In contrast, the metencephalon was much less infected than expected from its volume. Comprising ∼12% of the brain volume it contained only ∼5% of cysts. The remaining major brain parts (the diencephalon, mesencephalon and myelencephalon) contained numbers of cyst that corresponded well with their volumes.

**Table 1:** Quantitative data on *Toxoplasma gondii* cysts distribution.

Brain region	Volume (mm^3^)	Mean numbers of cysts ± SD	Mean cyst density ± SD (cysts/mm^3^)	Tropism index^a^	GLMM^b^
**Forebrain cortex**					
Olfactory bulb	11.09	38.8±23.76	3.50±2.14	5	***
Olfactory nucleus	2.19	6.8±6.62	3.11±3.03	3	n.s.
Frontal association cortex	1.31	7±4.69	5.33±3.57	4	***
Orbital cortex	4.24	13.8±7.98	3.26±1.88	5	***
Olfactory tubercle	1.62	3.4±4.92	2.10±3.05	2	n.s.
Piriform cortex	15.02	21.8±19.28	1.45±1.28	1	n.s.
Tenia tecta	1.14	6.6±4.45	5.79±3.91	4	***
Dorsal peduncular cortex	0.61	0.6±0.80	0.99±1.32	2	n.s.
Limbic cortex	1.93	2±1.79	1.04±0.93	2	n.s.
Cingulate cortex	4.45	9.6±7.68	2.16±1.73	3	n.s.
Motor cortex	16.14	51.4±45.94	3.17±2.85	5	***
Somatosensory cortex	28.45	78.6±70.31	2.76±2.47	5	***
Insular cortex	6.76	13.6±16,41	2.01±2.43	2	n.s.
Retrosplenial cortex	5.82	5.8±1.47	1.00±0.25	1	n.s.
Parietal cortex	1.61	3.8±2.93	2.36±1.82	3	n.s.
Visual cortex	11.24	25.8±21.83	2.30±1.94	4	n.s. (*)
Auditory cortex	5.22	8.4±7.17	1.61±1.38	3	n.s.
Temporal cortex	1.12	2.4±4.32	2.15±3.86	1	n.s.
Entorhinal cortex	12.65	39.4±24.51	3.12±1.94	5	***
Hippocampus	27.02	64.4±59.92	2.38±2.22	4	***
**Forebrain subcortical regions**					
Amygdala	9.59	20.6±15.33	2.15±1.60	4	n.s.
Septal nuclei	4.46	5.6±4.76	1.25±1.07	2	n.s.
Caudate putamen	20.92	17.4±6.09	0.83±0.29	0	***
Globus Pallidus	2.30	1±1.26	0.44±0.55	2	n.s. (*)
Nucleus accumbens	3.28	4.4±3.20	1.34±0.98	1	n.s.
Ventral pallidum	1.66	5.6±1.85	3.38±1.12	5	n.s. (**)
Substantia innominata	0.89	0.2±0.40	0.22±0.45	1	n.s.
Thalamus	16.58	32.2±15.93	1.94±0.96	3	n.s.
Hypothalamus	11.86	10.4±6.22	0.88±0.52	1	***
Zona incerta	1.56	3±1.41	1.93±0.91	3	n.s.
**Midbrain**					
Superior colliculus	4.59	7.6±9.46	1.66±2.06	2	n.s.
Inferior colliculus	6.26	6.4±6.05	1.02±0.97	1	n.s. (**)
Tegmentum	6.73	15±10.35	2.23±1.54	4	n.s.
Periaqueductal gray	2.83	3.6±5.24	1.27±1.85	1	n.s.
Substantia nigra	1.96	4.2±2.79	2.14±1.42	3	n.s.
Paranigral nucleus	0.07	0.6±1.20	8.11±16.22	1	n.s. (*)
Ventral tegmental area	0.72	3.2±3.66	4.45±5.08	3	*
Deep mesencephalic nucleus	4.49	5.2±4.12	1.16±0.92	2	n.s.
Lateral lemniscus nuclei	1.09	2.2±1.47	2.03±1.35	3	n.s.
**Hindbrain**					
Cerebellum	35.86	20.6±17.06	0.58±0.48	0	***
Pontine nuclei	0.75	0±0	0.00±0	0	***
Vestibular nuclei	2.29	3.8±2.04	1.66±0.89	3	n.s.
Locus coeruleus	0.08	0.2±0.40	2.59±5.18	1	n.s.
Periolivary region	1.19	1.8±1.33	1.51±1.12	3	n.s.
Raphe nuclei	1.87	2±1.26	1.07±0.68	2	n.s.
Intermediate reticular nucleus	1.89	4.2±4.40	2.23±2.33	3	n.s.
Parvicellular reticular nucleus	4.58	2.6±3.32	0.57±0.73	1	**
Gigantocellular reticular nucleus	3.34	7.2±4.62	2.16±1.39	4	n.s.
Spinal trigeminal nucleus	4.58	4±3.03	0.87±0.66	2	n.s.
Medullary reticular nucleus	0.59	5±4.90	8.48±8.30	4	***
Hypoglossal nucleus	0.45	2.6±3.38	5.83±7.58	3	**
Facial nucleus	0.76	3.4±4.92	4.50±6.51	2	*
**Fiber tracts & commissures**					
Corpus callosum	10.29	0.2±0.40	0.02±0.04	0	***
Cerebral peduncle	1.54	1.2±1.17	0.78±0.76	1	n.s. (*)
Anterior commissure	1.05	0±0	0.00±0	0	***
Capsula interna	2.43	0±0	0.00±0	0	***

a Tropism index indicates the number of animals in which infestation was higher than expected from the total parasite load and volume of a particular structure.

b The GLMM (generalized linear mixed model) tests positive or negative tropism toward a particular structure. Significance after Šidák correction is indicated by asterisks; significance before the Šidák correction is given in parentheses. Significance levels:

***, P<0.001;

**, P<0.01;

*, P<0.05; n.s., not significant. See Methods for details.

Within the telencephalon, a consistently high cyst density (i.e., seen in all five brains examined) was observed in the external plexiform layer of olfactory bulb, the ventral pallidum and the entorhinal, somatosensory, motor and orbital cortices. Moreover, in four out of five brains examined, a high cyst density was observed in the frontal association and visual cortices, the tenia tecta, and, importantly, the hippocampus and the amygdala. Among non-telencephalic brain regions, the tegmentum, the gigantocellular and medullary reticular nuclei exhibited a high cyst density in four out of five brains examined. By contrast, a consistently low incidence of tissue cysts was recorded in the cerebellum, the pontine nuclei, the caudate putamen, the accessory olfactory bulb, and virtually all compact masses of myelinated axons. For instance, the corpus callosum, the anterior and posterior commissures, the capsula interna, and the cerebellar peduncles were devoid of cysts; the cerebral peduncle, the fimbria of the hippocampus and the hippocampal commissures featured a low incidence of cysts. On average, the cyst density was ∼12 times lower in the white matter than that in the grey matter. In four out of five brains examined, a low cyst density was also observed in the piriform, retrosplenial and temporal cortices, the nucleus accumbens, the substantia innominata, the hypothalamus, the periaqueductal grey, the inferior colliculus and the parvicellular reticular nucleus.

It is notable that, in the isocortex and the cerebellar cortex, cysts were most abundant within the molecular and/or external granular layers and within the molecular layer, respectively.

### No evidence for tropism toward the dopaminergic system

None of the structures containing dopaminergic neurons exhibited a consistently high incidence of *T. gondii* cysts (Table 1, Figs. 4, 5). The substantia nigra (A 9) and the ventral tegmental area (A 10) were highly infected in three and two animals, respectively. In the remaining animals, these structures exhibited a relatively low incidence of cysts. In the zona incerta, a high incidence of cysts was observed in a single animal, a low incidence in two animals. Moreover, cysts were not localized within its ventromedial part that contains dopaminergic neurons (A 13, cf. Fig. 4). Likewise, the mesencephalic periaqueductal grey (A 11) was highly infected only in a single animal. Hypothalamus and, in particular, all hypothalamic regions containing dopaminergic neurons (i.e., dorsal and posterior hypothalamus, A 11; arcuate nucleus, A 12; periventricular hypothalamus, A 14; the ventral surface of hypothalamus and ventral part of the bed nucleus of stria terminalis, A 15) featured a low incidence of cysts (Figs. 4, 5). Finally, within the olfactory bulb, cysts were preferentially located in the external plexiform layer and only seldom occurred in the glomerular layer. Thus, the cysts were in close vicinity but not in contact with the dopaminergic periglomerular cells (note that both dendrites and axons of these interneurons are confined within the glomerular layer).

### No evidence for tropism toward the hypothalamic defensive system (HDS)

The HDS (for review see [37]) plays a pivotal role in eliciting innate defensive behavior after exposure to a natural predator. It consists of three highly interconnected hypothalamic medial zone nuclei, namely, the anterior hypothalamic nucleus, the dorsomedial part of the ventromedial nucleus and the dorsal premammillary nucleus. These nuclei were devoid of cysts.

In addition, except for the amygdala (see below) none of the brain regions that provide major neural inputs to and/or outputs from the HDS featured a consistently high incidence of *T. gondii* cysts. For instance, the bed nucleus of the stria terminalis, the infralimbic cortex and the lateral preoptic area were virtually devoid of cysts; the lateral septal nucleus and the periaqueductal grey was highly infected only in a single animal; the prelimbic cortex and the ventral tegmental area in two animals. Amygdaloid nuclei that project to the HDS, namely the posteroventral part of the medial amygdaloid nucleus and the posterior part of the basomedial amygdaloid nucleus were infected in three animals; the lateral amygdaloid nucleus that receives input from the HDS was infected in four animals.

Besides the aforementioned structures, numerous other brain regions have been reported to be activated by exposure to a live cat or cat odor [38]–[43]. None of these brain regions but the motor cortex featured a consistently high incidence of cysts. A few other regions also exhibited a high cyst density, although not in all animals. The anterior olfactory nucleus, the cingulate cortex, and the ventral orbital cortex were highly infected in three animals. The remaining telencephalic “defense-related” regions, including the infralimbic, piriform and retrosplenial agranular cortices, the septum, the shell of the nucleus accumbens and the caudate putamen were less infected than expected from their volumes. The lateral hypothalamus, the perifornical region and the reuniens thalamic nucleus were infected in three animals, the dorsomedial hypothalamic nucleus and the anteromedial thalamic nucleus in one animal. Other hypothalamic and thalamic “defense-related” regions, including the medial preoptic, lateral preoptic and retrochiasmatic areas, and the paraventricular hypothalamic and paraventricular thalamic, intralaminar thalamic and lateral habenular nuclei were devoid of cysts. Finally, the cuneiform nucleus was highly infected in one animal and a single cyst was observed in the locus coeruleus.

### No evidence for tropism toward specific amygdaloid compartments

As noted above, the amygdaloid complex was highly infected in four out of five brains examined. It is to be noted, however, that cysts were not preferentially associated with a particular amygdaloid compartment and the cyst distribution differed significantly between individuals. Nevertheless, if data compiled from five brains are considered, some amygdaloid divisions were more infected than others. A high cyst density was observed in the anterior amygdaloid area, the medial and cortical amygdaloid nuclei; a moderate cyst density in the lateral, basolateral and basomedial amygdaloid nuclei and in the amygdalohippocampal area. Importantly, a single cyst was observed in the central amygdaloid nucleus.

### Distribution of *T. gondii* cysts in the hippocampus and parahippocampal region

The entorhinal cortex was highly infected in all brains examined. Both the medial and lateral entorhinal cortices were highly infested with cysts. A much lower cyst density was observed in the ectorhinal and perirhinal cortices, which were infected in one and three animals, respectively. Similarly, the presubiculum and parasubiculum were infected only in two animals. The hippocampus was highly infected in four out of five brains examined. But again, distribution differed significantly between individuals and no tropism toward a particular hippocampal compartment was observed. The cysts were abundant within all cytoarchitectonically distinct regions: the dentate gyrus, the CA1–CA3 fields and the subiculum. Topographically, both the dorsal (septal) and the ventral (temporal) hippocampus were highly infected.

### Histopathological features of latent toxoplasmosis

**Figure 6:**
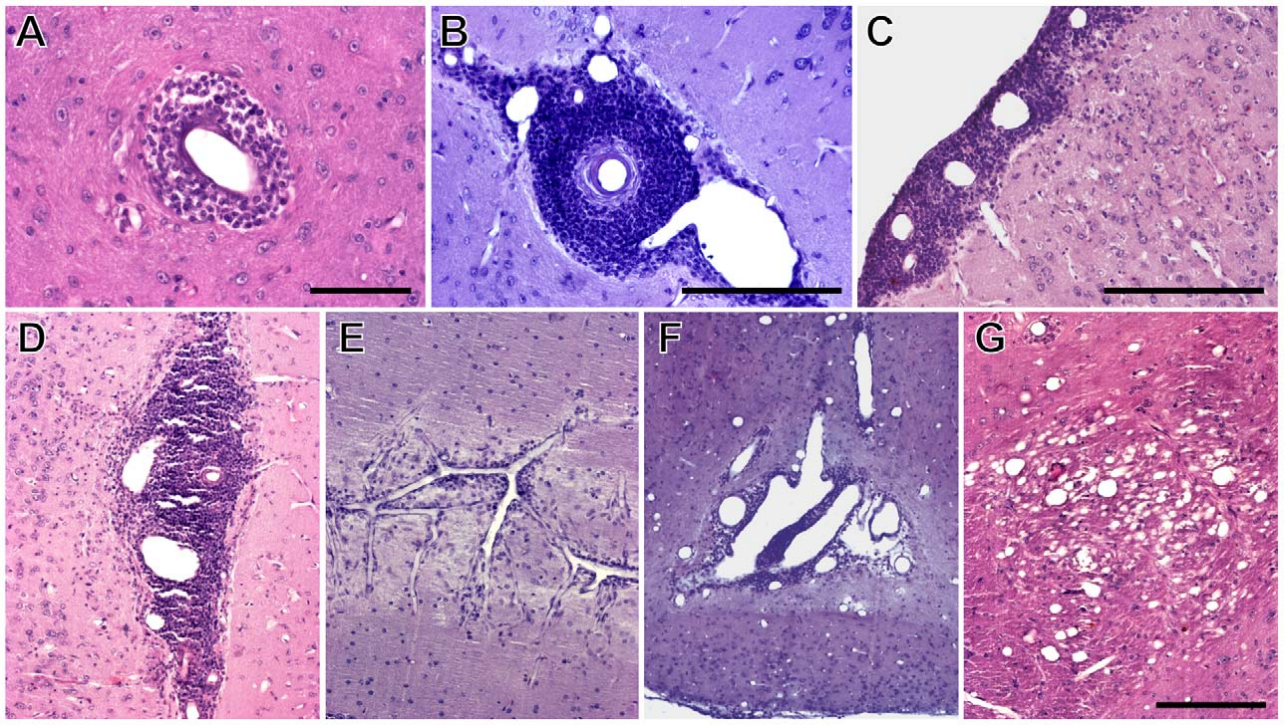
Histopathological lesions associated with latent toxoplasmosis. (A–E) Perivascular and leptomeningeal infiltrations of inflammatory cells. (A, B) Detail of perivascular cuffing. (C–E) Leptomeningeal and perivascular cuffing on the surface of cerebral cortex (C), in the interhemispheric region (D) and in the cerebellum (E). (F) Extensive necrosis in the hypothalamus (observed in one mouse). (G) Vacuolisation of brain parenchyma in the thalamus. Note that vessels were dilated *post mortem* by pressure of the perfusion liquids. Scales, 100 µm (A), 200 µm (B, C, G, in G for D–G).

Macroscopically, brains of the infected mice appeared normal in volume, shape and coloring. Nevertheless, a microscopic analysis revealed various histopathological lesions. Numerous perivascular and leptomeningeal infiltrations of inflammatory cells were observed, most often on the surface of the cerebral cortex and in the interhemispheric region (Figs. 6A–E); occasional occurrence of necrosis of the brain parenchyma was also noted (Figs. 6F, G). The motor, somatosensory, entorhinal, frontal association and orbital cortices were most affected, but the histopathological lesions were also seen in the olfactory bulb, the hippocampus, the thalamus, the hypothalamus, the caudate putamen and the cerebellum. Importantly, these lesions were not located in the close vicinity of *T. gondii* cysts.

The cerebral microvasculature exhibited strong dilatation. However, the omnipresence and the extent of vasodilatation strongly suggest that vessels were dilated *post mortem* by pressure of the perfusion liquids.

None of the above described lesions but vasodilatation were observed in the control mice.

## Discussion

Cysts containing *T. gondii* were found in brains of all the infected mice, but the total parasite load differed starkly between experimental animals. Remarkably, the most infested brain exhibited a more than one order of magnitude higher cyst density than the least infested one. The cysts were distributed in a wide variety of brain regions. Indeed, as much as 92% of the brain regions examined contained tissue cysts. Hence, *T. gondii* might affect the information processing within a wide variety of brain functional systems, provided that the parasite is able to alter brain function locally (no matter by which proximate mechanism). In spite of a significant inter-individual variability, four general properties of *T. gondii* distribution stand out from the scrutiny performed here. First, the cerebral cortical areas almost always exhibited higher cyst density than subcortical regions. Notable exceptions comprised of the poorly infested piriform, retrosplenial and temporal association cortices on one hand and the highly infested amygdala and tegmentum on the other hand. Second, the cerebellum featured a consistently low incidence of cysts. Third, compact myelinated fiber tracts and commissures were virtually devoid of cysts. Moreover, subcortical structures bounded by compact masses of myelinated axons (e.g., the caudate putamen and the pontine nuclei) were ranked among the least infected structures, implying that compact myelinated bodies constitute a barrier for the parasite. Finally, a selective tropism of *T. gondii* toward a particular functional system was not observed. In the context of the recently discussed hypotheses (see Introduction) it is important to note that the parasite is guided neither toward the dopaminergic system nor toward the hypothalamic defensive system.

### Health status and total parasite load in the brain

The clinical appearance of acute acquired toxoplasmosis depends on the virulence of the *T. gondii* strain used, the initial dose and the port of entry of the parasite, the host species and/or strain, and the immunological and genetic status of the host [44]. To mimic natural conditions, peroral inoculation and a low challenge dose were used in the current study. We observed no apparent symptoms of the acute toxoplasmosis except for transient reduction of the body weight, which is in agreement with the previously reported asymptomatic course of the HIF strain infection [20], [45]. Weight reduction during the fourth week p.i. probably coincided with the onset of cellular and antibody immune responses and formation of intracellular cysts in the brain [46], [47]. A mild loss of weight in control mice immediately after the inoculation could be caused by irritation of the digestive tract with the stomach tube.

The aforementioned factors are likely also decisive for the parasite load in the brain, which, in turn, determines behavioral phenotype of the host with latent toxoplasmosis. Studies addressing behavioral and/or neurological abnormalities induced by *T. gondii* infection utilized various experimental paradigms, including intraperitoneal (e.g., [7], [9], [10], [21], [31], [32], [48], [49]), subcutaneous (e.g., [17], [29], [30]) or peroral (present study, [6], [19], [20], [25], [45], [50]) administration of various parasite doses and congenital *T. gondii* infection [18], [23], [51], [52].

Not unexpectedly, the different paradigms yielded ample variation in brain infestation (cf. e.g., current study, [7], [17], [20], [51]). More surprisingly, stark differences in brain cyst numbers were observed even when the same *T. gondii* strain and the administration protocol were utilized. For instance, the peroral inoculation of 10 tissue cysts of the HIF strain resulted in high (∼770 cysts per brain), mid (∼225 cysts per brain) and low (∼46 cysts per brain) brain infestation in CD1 outbred mice, CBA/J mice and F1 crosses between BALB/c and B10A mice, respectively (present study, [20], [45]). These observations are in line with strain-dependent differences in the murine susceptibility to toxoplasmosis [53], [54]. A rather low brain infestation has been reported in the rat [7], [32].

### Lack of tropism and probabilistic nature of T. gondii distribution

The present study and earlier reports on the parasite distribution in the brain ([7], [21], [28]–[32], see Introduction) point to some general features but also to a marked variability in the parasite distribution patterns. First, and foremost, a very selective tropism of *T. gondii* toward a specific brain region and/or functional system is not indicated. All the aforementioned studies describe a rather wide *T. gondii* distribution. Moreover, the observed distribution patterns differ between experiments. For instance, the highest cyst density was reported for the olfactory bulb [29], the olfactory bulb and the cerebral cortex (present study), the cerebral cortex [31], the amygdala [7], the nucleus accumbens and the ventromedial hypothalamus [32] and the medulla oblongata [30]. In rat, the parasite seems to be much more confined to the medial brain regions than in the mouse (cf., [32]). Likewise, the olfactory bulb seems to be much less infected in the rat [7] when compared to the mouse. In AIDS patients with *toxoplasma encephalitis*, the lesions were found mainly in hemispheres (often in the fronto-parietal cortex), thalamus and basal ganglia [33]–[35], the parasites were scattered throughout the different brain parts [36]. These discrepancies may hypothetically be attributed to the differences in host species and/or strain, the *T. gondii* strain used, the inoculation paradigm and the post-infection time. One may also speculate that at least some of the aforementioned findings are inaccurate since prior studies are either based on qualitative analyses or semi-quantitative analyses of arbitrarily chosen sections or restricted to brain regions of interest. In any case, this limited data set suggests an unforeseen variability in the parasite distribution.

Second, a low incidence of the parasite in the cerebellum has been reported repeatedly (present study, [21], [30], [31]). The low level of infection might be caused by the cerebellar cytoarchitecture. The cerebellum features an extremely high cellular density, very small neurons and a low glia to neuron ratio [55], [56]. Given that neurons are much less efficiently infected by *T. gondii* than are astrocytes, and that cerebellar granular neurons are still less efficiently infected in vitro than hippocampal neurons (for review, see [57]), it is tempting to speculate that a high proportion of small, tightly packed granular neurons constitutes a limiting factor for *T. gondii* zoites proliferation and subsequent cyst formation.

Third and finally, a low incidence of cysts is also typical for the white matter ([31], present study). The present study highlights a virtual absence of cysts in compact myelinated fiber tracts and commissures. This observation is in line with the fact that *toxoplasma encephalitis* is, in contrast to encephalitis of other etiology (e.g. primary brain lymphoma), rarely associated with lesions in the corpus callosum [58].

Taken together, the *T. gondii* distribution in the brain seems to be of a probabilistic nature. Factors determining the parasite distribution remain largely unclear. As noted above, we suggest that compact myelinated bodies may act as natural barriers for the parasite and that cytoarchitectural features such as the neuron size and glia to neuron ratio may influence the local parasite load. However, it seems likely that the mechanism underlying brain colonization during the acute infection is most decisive for the parasite distribution. *T. gondii* tachyzoites disseminate by the bloodstream using dendritic cells and monocytes/macrophages as transporters (Trojan horses) to the organ tissues, including the brain extravascular space ([59], [60], and citations therein). Abundant presence of *T. gondii* in the brain parenchyma and its scarcity in the plexus choroideus and circumventricular regions ([31], [61], present study, but see [30]) suggest that the parasite crosses the blood-brain barrier via the cerebral microvasculature rather than the blood-cerebrospinal fluid barrier via the plexus choroideus. Furthermore, it has been suggested that a higher number of parasites may reach brain regions that are characterized by higher blood flow during the acute phase of infection [31]. Although the hemodynamic response to neural activity is proximately driven by neuronal signalling, the local blood flow generally correlates with metabolic activity [62]. Consistently with this “high blood flow – high parasite density” hypothesis, we observed a high incidence of the parasite in the olfactory bulb and primary visual, somatosensory and motor cortices that feature a high level of oxidative metabolism, but a rather low incidence of parasite in the piriform cortex and some association cortices that feature a moderate level of oxidative metabolism (c.f. [63]). On the other hand, we observed accumulation of the parasite in neither the retrosplenial and auditory cortices nor the subcortical regions that feature a high metabolic activity. So this hypothesis is not tenable. Consequently, other factors, e.g. local properties of the blood-brain barrier, seem to be at work. A firm understanding of *T. gondii* distribution in the brain will require further mechanistic studies.

### Effective manipulation of host behavior without well targeted tropism of parasite?

Recent literature suggests that *T. gondii* highly specifically decreases the vigilance of rats and mice for feline predators [6]–[9]. Infected rodents lose their innate fear and in fact are mildly attracted to cat odor, but retain the aversion to odors of other predators like mink or dog. This ‘suicidal’ attraction thus appears to be restricted to the feline predators, which serve as definitive hosts of the parasite. Such an intriguingly specific manipulation of the host behavior clearly calls for a highly specific mechanistic explanation [5]. A selective impairment of olfaction and an alteration in the emotional valence of cat odor achieved by the *T. gondii*-induced modification of neural processing within the amygdala and/or the nucleus accumbens are most discussed in this context (for review see [5], [13]). While all these hypotheses are highly relevant, their general validity is undermined by the lack of well targeted tropism of *T. gondii*. Indeed, the consistency in the behavioral responses starkly contrasts with the differences in the total parasite load and distribution between individuals, species and, in particular, between different studies (see discussion above). For instance, a high parasite density in the olfactory bulb has been reported in the mouse (∼3.5 cyst/mm^3^, present study) but not in the rat (<0.1 cyst/mm^3^, [7]). Furthermore, the distribution of the parasite does not suggest that *T. gondii* targets specifically the olfactory glomeruli important for processing innate responses to the predator odor. Innate responses to aversive odorants, including the avoidance of predators' smells, are mediated by receptors located in a dorsal zone of the olfactory epithelium, which project their axons to the anterodorsal domain of the olfactory bulb [64]. In addition, at least some aspects of feline odor are perceived and processed as pheromone-like stimuli that activate the accessory olfactory bulb and its projection areas [41], [43]. One would thus expect tropism towards the dorsal domain of the main accessory olfactory bulb and towards the accessory olfactory bulb. However, we have observed *T. gondii* cysts throughout the olfactory bulb, with the highest density within its lateral portion and almost no cysts in the accessory olfactory bulb.

Likewise, subtle tropism towards the amygdala has been reported in both rat and mouse, but the absolute density is much higher in the mouse (rat ∼0.15 cyst/mm^3^, [7]; mouse ∼2.1 cyst/mm^3^, present study); the parasite is not associated with particular, functionally well-defined amygdaloid nuclei (present study). The amygdala is not only essential for Pavlovian fear conditioning (for review, see e.g. [65], [66]) but via projection to the HDS also modulates unconditioned (innate) defensive responses [37]. Receiving inputs form thalamic and cortical sensory processing regions [67] it seems to be important for detecting and assessing danger in the sensory world. It is notable in this context that we have observed a rather high parasite density within the medial amygdaloid nuclei, which receive a robust input from the main and accessory olfactory systems [67] and express c-Fos after cat-odor exposure [39]. Lesions of the medial amygdala reduce freezing and risk assessment during cat-odor exposure [68]. It is also important to note that we have observed a high parasite density (∼2.4 cyst/mm^3^) in the ventral hippocampus. The ventral hippocampus modulates unconditioned defensive behaviors through its connections with the amygdala and hypothalamus (e.g., [69]–[71]). It sends projections to the HDS via the amygdala and bed nucleus the stria terminalis, and via the lateral septum. Lesions of the ventral hippocampus reduce anxiety-like behaviors in various experimental paradigms (e.g., [72]–[74]), importantly including cat-odor exposure [75]. But again, the ventral hippocampus was poorly infested in rat (<0.1 cyst/mm^3^, [7]). Finally, a high relative parasite density in the nucleus accumbens has been reported in the rat [32] but not in the mouse (∼1.3 cyst/mm^3^, present study). Indeed, this structure was less infected than expected from its volume in the mouse. Besides a pivotal role in encoding reward and aversion [76], the nucleus accumbens seems to be also involved in fear conditioning [77]. The activity of its principal neurons – medium spiny GABAergic neurons – is modulated by dopamine input from the ventral tegmental area and glutamate input from regions including the prefrontal cortex, amygdala and hippocampus (for review, see [76]), i.e., regions all reported to be infested in rodents with latent toxoplasmosis ([7], [32]; present study). Moreover, recent work suggests that changes in the activity of the dopaminergic neurons in the ventral tegmental area can also encode both rewarding and aversive states [78].

Taken together, the present data suggest that the attenuated defensive behavior of infected rats and mice may stem from serendipitous infestation of various brain regions implicated in modulation of defensive and aversive behaviors. These regions include the amygdala, the ventral hippocampus, the nucleus accumbens, the ventral tegmental area and the medial prefrontal cortex. The probability that at least some of these regions will be infected by chance seems to be quite high. This is consonant with reports that *T. gondii* reduces anxiety (the elevated plus-maze and the social interaction tests – [32]; the hole-board test – [20]) and affects learned fear response in chronically infected rodents [48]. Furthermore, it is also conceivable that *T. gondii* alters the olfactory processing of cat odor through serendipitous infestation of the olfactory glomeruli processing this information. Nevertheless, the predator-specific reduction of vigilance (i.e., fatal attraction to cat odor) is difficult to explain. While a possible existence of neural mechanisms or substrates that are dedicated only to the processing of cat odors (c.f., [5]) clearly deserves further research, the above reviewed observations are inconsistent with a notion that the parasite specifically targets the neural substrate of innate aversion to felines.

### A potential effect of *T. gondii* on motor coordination, sensory processing and spatial navigation

A disruption of the defensive behavior clearly is not the only mechanism by which *T. gondii* can increase its transmission to the definitive feline host. First and foremost, infestation of various stages of the somatosensory and motor systems may compromise motor performance, sensorimotor integration and motor coordination. Indeed, we have observed a high parasite density in the somatosensory and motor cortices and in the ventrolateral thalamic nucleus. The latter is the main motor thalamic relay conveying cerebellar input to the motor and premotor cortical areas; the cerebello-thalamo-cortical pathway is most likely involved in the coordination of multi-joint movements (for review, see [79]). *T. gondii* infestation of the spinal cord has also been reported recently [28]. These observations are in line with motor coordination deficits reported in infected rodents [10], [21], [22], [80] and humans [81]–[83].

Second, a high to medium infestation of the olfactory bulb, the anterior olfactory nucleus, the visual cortex and the barrel field of the primary somatosensory cortex (i.e., the region that processes tactile information from the whiskers) may affect processing of sensory information. While nothing is known about the sensory capacities of chronically infected rodents, deficits in sensory driven reflexes such as the visual placing response (an extension of the paws on approach to a visual surface) or orientation response to whisker stimulation [21] clearly demonstrate impairment of sensorimotor integration. In addition, reduced sniffing, rearing and whisking during the first exposure to an open field [10], [20], [21], [84] indicate sensory attention deficits. A prolonged time devoted to sniffing and head dipping in holes in the hole-board test [20] seems to contradict these results. However, it is likely the anxiolytic effect of toxoplasmosis what accounts for high scores in this exploration-based anxiety test (c.f., [85]). Moreover, it is tempting to speculate that longer sniffing at holes is required to assess olfactory information due to *T. gondii*-induced impairment of olfaction. But even if true, the impairment clearly is not an absolute all-or-nothing phenomenon since infected rodents reportedly retain an aversion to food with a novel odor [7] and aversion to odors of predators that do not serve as a definitive host of *T. gondii* [8], [9].

Finally, a high infestation of the entorhinal cortex (∼3.1 cyst/mm^3^) and the dorsal hippocampus (∼2.4 cyst/mm^3^) observed in this study suggests that *T. gondii* may reduce the efficiency of near space navigation (i.e., orientation within home range). These structures play pivotal roles in spatial navigation [86]–[88] and certain forms of learning and memory [71], [89], [90]. More specifically, the hippocampal place cells (pyramidal neurons) exhibit location-specific activity and their entire population likely generates an abstract map-like representation of the animal's spatial surroundings [86]. Besides encoding the animal's position, they are involved in the forming and consolidation of context-specific episodic memory (e.g., [91]–[93]). The entorhinal cortex harbors the grid cells [94], [95], which possess tessellating firing fields and encode environment-independent (path integration generated) neuronal map of self-location [88], and head direction cells [96], which have direction-specific activity and encode the animal's directional heading [97]. Thus, *T. gondii* is well placed to affect spatial orientation, learning and memory. Yet, behavioral evidence remains controversial. Infected mice exhibited diminished spatial learning and memory in double-training maze experiments [49], [98] and in the 8-arm radial maze test [50], but normal memory in object placement and object recognition tests [21]. Y-maze experiments suggested deficits in spatial working memory but not in recognition memory [9]. Infected rats showed impaired spatial learning but not memory in the same experiments [49], [98] and intact spatial learning in the Morris water maze [7]. It is likely, however, that various parasite loads and distributions in the brain account for most of these inconsistencies. Mice often feature a much higher brain infestation than rats (see discussion above) and hence more severe cognitive deficits. The fact that the learning performance of mice is negatively correlated with the number of brain cyst [49] further supports this assumption. Additionally, in stark contrast with the situation in mice, the hippocampus and parahippocampal region were reported to be poorly infested or devoid of cysts in rats [7], [32]. The histological findings thus seem to be largely consistent with the cognitive deficits.

### Mechanisms underlying T. gondii-induced modification of brain function

As we have argued above, the pattern of *T. gondii* distribution in the brain may explain many of the behavioral abnormalities observed in the chronically infected rodents. But how does *T. gondii* alter brain function locally? The proximate mechanisms underpinning the *T. gondii* effect on local neural processing were not the subject of the present study. Nevertheless, two important inferences as to the mechanism of action can be drawn from the distribution data presented here. First, a remarkable interindividual variability in the distribution of the observed histopathological lesions suggests that *T. gondii*-induced local inflammatory immune responses and neurodegenerations cannot account for the highly consistent and specific behavioral changes associated with latent toxoplasmosis. However, it has to be noted for the sake of unbiased interpretation that frequent occurrence of perivascular inflammation contiguous to the hippocampus and the aqueduct of Sylvius has been reported recently [10]. In any case, altered neuromodulator levels secondary to inflammation may influence the behavioral phenotype of infected individuals. This phenomenon, together with the neurodegenerative loss of brain parenchyma, might significantly increase the predation risk in the infected rodents. Second, the lack of tropism towards the dopaminergic system clearly shows that the parasite is not specifically guided to dopaminergic neurons. On the other hand, the high average cyst densities in the ventral tegmental area (∼4.4 cyst/mm^3^) and the substantia nigra (∼2.1 cyst/mm^3^) suggest that the mere serendipitous infestation of the regions containing dopaminergic neurons may have a significant effect on dopamine metabolism. This is in line with the recent evidence for increased dopamine release from dopaminergic neurons infected with *T. gondii* [16]. Moreover, the fact that *T. gondii* cysts in the mouse brain co-express high levels of the parasite-encoded, dopamine synthesis enzyme tyrosine hydroxylase and dopamine itself raise an intriguing possibility that the parasite can synthesize and release dopamine and thereby increase the dopamine levels also outside of the dopaminergic neurons [15], [16]. Since dopamine receptors are rather ubiquitous (see e.g., [99]), the parasite-mediated increase of local dopamine levels may well provide a mechanism for altered information processing within many of the above discussed brain regions containing *T. gondii* cysts, including the olfactory bulb, the amygdala, the hippocampus, the nucleus accumbens and several cortical and thalamic regions.

### Conclusion

The results of the present study show that *T. gondii* cysts are distributed throughout the brain and the parasite is not guided toward a particular functional system. The striking interindividual differences in the total parasite load and cyst distribution indicate a probabilistic nature of brain infestation. However, the cyst density is not homogeneous; some brain regions are consistently more infected than others. We argue that factors underlying brain colonization during acute infection, including local properties of the blood-brain barrier, regional cytoarchitectural features, the level of metabolism and blood flow, and the presence of compact myelinated bodies as natural barriers for the parasite, can contribute interactively to the increased infestation of certain cortical and subcortical regions. We further argue that subtle tropism stemming from uneven brain colonization suffices for the explanation of the numerous behavioral abnormalities observed in the chronically infected rodents, provided that the parasite is able to alter brain function locally. Specifically, the infestation of the somatosensory and motor cortices, and the ventrolateral thalamic nucleus may account for motor and coordination deficits; the infestation of the olfactory bulb, the visual cortex and the barrel field of the somatosensory cortex for sensory attention deficits and compromised sensorimotor integration; the infestation of the entorhinal cortex and the dorsal hippocampus for cognitive deficits and compromised spatial orientation; the infestation of the amygdala, the ventral hippocampus, the nucleus accumbens and the ventral tegmental area for the attenuated defensive behavior and decreased vigilance for predators, respectively. Thus, equipped with a capacity to modulate local dopamine metabolism, *T. gondii* can effectively change the behavioral phenotype of infected hosts despite the absence of well targeted tropism.

## Materials and Methods

### Ethics statement

Animal husbandry and all experimental procedures complied with the European Community regulations on the care and use of experimental animals, and were approved by the institutional animal care and use committee of the Faculty of Science, Charles University in Prague (2004/10).

### Parasites and experimental animals

Fifty-eight male mice of the CD1 outbred strain, obtained from Anlab (Czech Republic), were used. The mice were housed in plastic cages with wood shaving bedding and nesting material and kept in a 12∶12 h light-dark cycle. Food and water were available *ad libitum*. At 6 months of age, twenty-nine mice were inoculated per orally with 0.5 ml of brain suspension in saline, which contained 10 tissue cysts of the avirulent cyst-forming HIF strain of *Toxoplasma gondii*, isolated in 1993 in the Czech Republic from the cerebrospinal fluid of a male HIV-positive patient with asymptomatic toxoplasmosis [45]. The brain suspension was prepared from the brain of a mouse infected previously and was administered to mice by a stomach tube. A control group of twenty-nine sham infected mice received 0.5 ml of saline. All mice were regularly observed for symptoms of acute toxoplasmosis and their weights were recorded.

### Serological tests

Serological tests for toxoplasmosis were carried out in the National Reference Laboratory for Toxoplasmosis of the National Institute of Public Health, Prague. Blood for serological tests was taken from the orbital sinus of all experimental and control animals. Antibody titers for toxoplasmosis were determined by the complement fixation test (CFT) (SEVAC, Prague) according to standard procedure recommended by the manufacturer [100].

### Histology

Eighteen weeks after inoculation, five infected and one control mouse were deeply anesthetized with intraperitoneal injection of ketamine (150 mg/kg) and xylazine (15 mg/kg), and perfused with 50 ml of warm heparinized saline, followed by 50 ml of 4% Bouin's fixative. Brains were immediately dissected, postfixed for 3 days in the same fixative, embedded in paraffin and sectioned in the coronal plane at a thickness of 8 µm. Sections were mounted on subbed slides, stained with hematoxylin-eosin and coverslipped with DPX (Fluka, Buchs, Switzerland). Brains of other 7 infected mice were homogenized and the number of brain cysts was determined in ten samples of 10 µl suspension per each brain homogenate at 200× magnification.

### Data analysis

**All sections (i.e., ∼1600 sections per animal)** were systematically examined with an Olympus BX51 microscope at 200× magnification. The exact position of all tissue cysts and histopathological changes were noted; the cyst positions were drawn into coronal diagrams of a mouse brain [101] using CorelDraw 12 (Corel Corporation, Ottawa, Ontario, Canada). Cyst counts and densities were determined for 56 brain structures, total cyst counts were calculated for 5 fundamental brain parts.

Measurements of brain structure volumes were performed using a stereotaxic mouse brain atlas [101]. One hundred sixty one coronal diagrams were used for planimetry on a digitizer tablet; areas were measured using Scion Image for Windows (Scion Corporation, Frederick, Maryland, USA). The resulting area values were multiplied by the distance between the diagrams to arrive at the serial volumes. The sum of the volumes for a given structure was used to assess its relative size (see Table 1).

### Statistical methods

Data were analyzed using SPSS for Windows (version 16). A general linear model (GLM) repeated measures procedure was used to compare the changes in body weight after inoculation between infected and sham infected animals. The Kendall tau rank correlation coefficient was used to assess association between the numbers of brain tissue cysts and *T. gondii* antibody titers. A generalized linear mixed model (GLMM) with Poisson error structure and a logarithmic link function was used to test the null hypothesis that the cyst density is determined solely by the total parasite load and independent of the brain region. After the rejection of this hypothesis, we used the Kendall's coefficient of concordance to arbitrate whether the probability of a high or low infestation of a particular brain region is consistent among the infected mice. The GLMM was also used to test whether a particular brain region is infested more or less than expected from its volume. The Šidák correction was used to counteract the problem of multiple comparisons. Because the GLMM is prone to produce false positive results in small structures (due to accidental focal infestation, see e.g., the facial and hypoglossal nuclei), we also calculated a tropism index indicating the number of animals, in which infestation was higher than expected.

## Supporting Information

Abbreviations S1 **List of abbreviations.**
(DOCX)
