# Supplementary material for: The Distribution of Toxoplasma gondii Cysts in the Brain of a Mouse with Latent Toxoplasmosis: Implications for the Behavioral Manipulation Hypothesis
Source: PLoS One. 2011 Dec 14;6(12):e28925. doi: 10.1371/journal.pone.0028925 (PMC3237564; doi:10.1371/journal.pone.0028925)
Supplement: Abbreviations S1 — List of abbreviations. (DOCX) [file pone.0028925.s001.docx]

**List of Abbreviations:**

1Cb 1st Cerebellar lobule

2Cb 2nd Cerebellar lobule

3Cb 3rd Cerebellar lobule

3N oculomotor nucleus

3PC oculomotor nucleus, parvicellular part

3V 3rd ventricle

4Cb 4th Cerebellar lobule

4n trochlear nerve

4V 4th ventricle

5Cb 5th Cerebellar lobule

6Cb 6th Cerebellar lobule

6N abducens nucleus

7Cb 7th Cerebellar lobule

7n facial nerve

7N facial nucleus

8Cb 8th Cerebellar lobule

8n vestibulocochlear nerve

9Cb 9th Cerebellar lobule

10Cb 10th Cerebellar lobule

10N dorsal motor nucleus of vagus

11n root of accessory nerve

11N accessory nerve nucleus

12N hypoglossal nucleus

A1 A1 noradrenaline cells

A2 A2 noradrenaline cells

A5 A5 noradrenaline cells

A8 A8 dopamine cells

A13 A13 dopamine cells

AAD anterior amygdaloid area, dorsal part

AAV anterior amygdaloid area, ventral part

aca anterior commisure, anterior part

AcbC accumbens nucleus, core

AcbSh accumbens nucleus, shell

aci anterior commisure , intrabulbar part

ACo anterior cortical amygdaloid nucleus

acp anterior commisure, anterior part

Acs6/7 accessory abducens/facial nucleus

AD anterodorsal thalamic nucleus

ADP anterodorsal preoptic nucleus

AHA anterior hypothalamic area, anterior part

AHiAL amygdalohippocampal area, anterolateral part

AHiPM amygdalohippocampal area, posteromedial part

AID agranular insular cortex, dorsal part

AIP agranular insular cortex

AIV agranular insular cortex, ventral part

alv alveus of the hippocampus

AM anteromedial thalamic nucleus

Amb ambiguus nucleus

AMV anteromedial thalamic nucleus, ventral part

Ang angular thalamic nucleus

AOD anterior olfactory nucleus, dorsal part

AOL anterior olfactory nucleus, lateral part

AOM anterior olfactory nucleus, medial part

AOV anterior olfactory nucleus, veltral part

AP area postrema

APir amygdalopiriform transition area

apmf ansoparamedian fissure

APT anterior pretectal nucleus

APTD anterior pretectal nucleus, dorsal part

Aq aqueduct

ArcLP arcuate hypothalamic nucleus, lateroposterior part

ArcMP arcuate hypothalamic nucleus, medial posterior part

AStr amygdalostriatal transition area

Au1 primary auditory cortex

AuD secondary auditory cortex, dorsal area

AuV secondary auditory cortex, ventral area

AVDM anteroventral thalamic nucleus, dorsomedial part

AVPe anteroventral periventricular nucleus

AVVL anteroventral thalamic nucleus, ventrolateral part

B basal nucleus

Bar Barrington's nucleus

BIC nucleus of the brachium of the inferior colliculus

bic brachium of the inferior colliculus

BLA basolateral amygdaloid nucleus, anterior part

BLP basolateral amygdaloid nucleus, posterior part

BLV basolateral amygdaloid nucleus, ventral part

BMA basomedial amygdaloid nucleus, anterior part

BMP basomedial amygdaloid nucleus, posterior part

bp brachium pontis

bsc brachium of the superior colliculus

BSTIA bed nucleus of stria terminalis, intraamygdaloid division

BSTLD bed nucleus of stria terminalis, lateral division, dorsal part

BSTLJ bed nucleus of stria terminalis, lateral division, juxtacapsular part

BSTLP bed nucleus of stria terminalis, lateral division, posterior part

BSTMV bed nucleus of stria terminalis, medial division, ventral part

BSTLV bed nucleus of stria terminalis, lateral division, ventral part

BSTMA bed nucleus of stria terminalis, medial division, anterior part

BSTMPL bed nucleus of stria terminalis, medial division, posterolateral part

BSTS bed nucleus of stria terminalis, supracapsular part

C1 C1 adrenaline cells

CA1 field CA1 of hippocampus

CA2 field CA2 of hippocampus

CA3 field CA3 of hippocampus

cbc cerebellar commissure

cc corpus callosum

CC central canal

CeC central amygdaloid nucleus, capsular part

CeCV central cervical nucleus

CeL central amygdaloid nucleus, lateral division

CeM central amygdaloid nucleus, medial division

CeMPV central amygdaloid nucleus, medial posteroventral part

cg cingulum

Cg/RS cingulate/retrosplenial cortex

Cg1 cingulate cortex, area 1

Cg2 cingulate cortex, area 2

CGA cetral gray, alpha part

CGPn central gray of the pons

Cl claustrum

CL centrolateral thalamic nucleus

CM central medial thalamic nucleus

CnF cuneiform nucleus

Cop copula of the pyramis

cp cerebral peduncle, basal part

CPu caudate putamen

Crus1 crus 1 of the ansiform lobule

Crus2 crus 2 of the ansiform lobule

csc commissure of the superior colliculus

cu cuneate fasciculus

Cu cuneate nucleus

CxA cortex-amygdala transition zone

D3V dorsal 3rd ventricle

DC dorsal cochlear nucleus

DEn dorsal endopiriform nucleus

df dorsal fornix

DG dentate gyrus

dhc dorsal hippocampal commisure

DI dysgranular insular cortex

Dk nucleus of Darkschewitsch

DLG dorsal lateral geniculate nucleus

DLL dorsal nucleus of the lateral lemniscus

DLO dorsolateral orbital cortex

DLPAG dorsolateral periaqueductal gray

DM dorsomedial hypothalamic nucleus

DMPAG dorsomedial periaqueductal gray

DMSp5 dorsomedial spinal trigeminal nucleus

DP dorsal peduncular cortex

DpG deep gray layer of the superior colliculus

DpGi dorsal paragigantocellular nucleus

DpMe deep mesencephalic nucleus

DPO dorsal periolivary region

DpWh deep white layer of the superior colliculus

DRD dorsal raphe nucleus, dorsal part

DRI dorsal raphe nucleus, interfascicular part

DRV dorsal raphe nucleus, ventral part

DRVL dorsal raphe nucleus, ventrolateral part

dsc dorsal spinocerebellar tract

DTgC dorsal tegmental nucleus, central part

DTgP dorsal tegmental nucleus, pericentral part

dtgx dorsal tegmental decussation

DTM dorsal tuberomammillary nucleus

DTT dorsal tenia tecta

ec external capsule

ECIC external cortex of the inferior colliculus

Ect ectorhinal cortex

ECu external cuneate nucleus

eml external medullary lamina

EMi epimicrocellular nucleus

E/OV ependymal end subendymal layer /olfactory ventricle

EPl external plexiform layer of the olfactory bulb

exc extreme capsule

EW Edinger-Westphal nucleus

f fornix

FI flocculus

FC fasciola cinereum

FF fields of Forel

fi fimbria of the hippocampus

fmi forceps minor of the corpus callosum

fmj forceps major of the corpus callosum

fr fasciculus retroflexus

FrA frontal association cortex

Fu bed nucleus of stria terminalis, fusiform part

gcc genu of the corpus callosum

Gem gemini hypothalamic nucleus

Gi gigantocellular reticular nucleus

GiA gigantocellular reticular nucleus, alpha part

Gl glomerular layer of the olfactory bulb

Gr gracile nucleus

GrA granule cell layer of the accessory olfactory bulb

GrDG granular layer of the dentate gyrus

GrO granular cell layer of the olfactory bulb

Gus gustatory thalamic nucleus

hbc habenular commisure

HDB nucleus of the horizontal limb of the diagonal band

hf hippocampal fissure

I intercalated nuclei of the amygdala

IAD interanterodorsal thalamic nucleus

ic internal capsule

ICL intercrural fissure

ICj island sof Calleja

ICjM island sof Calleja, major island

icp inferior cerebellar peduncle

IF interfascicular nucleus

IG indusium griseum

IGL intergeniculate leaf

IL infralimbic cortex

ILL intermediate nucleus of the lateral lenmiscus

IMA intramedullary thalamic area

IMD intermediodorsal thalamic nucleus

In intercalated nucleus of the medulla

InC interstitial nucleus of Cajal

InCG interstitial nucleus of Cajal, greater part

InCo intercollicular nucleus

InG intermediate gray layer of the superior colliculus

IntDL interposed cerebellar nucleus, dorsolateral hump

IntP interposed cerebellar nucleus, posterior part

InWh intermediate white layer of the superior colliculus

IOB inferior olive, subnucleus B of the medial nucleus

IOBe inferior olive, beta subnucleus

IOC inferior olive, subnucleus C of the medial nucleus

IOD inferior olive, dorsal nucleus

IOK inferior olive, cap of Kooy of the medial nucleus

IOPr inferior olive, principal nucleus

IOVL inferior olive, ventrolateral protrusion

IPAC interstitial nucleus of the posterior limb of the anterior commissure

IPACL interstitial nucleus of the posterior limb of the anterior commissure, lateral part

IPACM interstitial nucleus of the posterior limb of the anterior commissure, medial part

IPC interpeduncular nucleus, caudal subnucleus

IPDL interpeduncular nucleus, dorsolateral subnucleus

IPDM interpeduncular nucleus, dorsomedial subnucleus

IPF interpeduncular fossa

IPl interpeduncular nucleus, intermediate subnucleus

IPL interpeduncular nucleus, lateral subnucleus

IPR interpeduncular nucleus, rostral subnucleus

IRt intermediate reticular nucleus

LA lateroanterior hypothalamic nucleus

La lateral amygdaloid nucleus

LaDL lateral amygdaloid nucleus, dorsolateral part

LaVL lateral amygdaloid nucleus, ventrolateral part

LaVM lateral amygdaloid nucleus, ventromedial part

LC locus coeruleus

LDDM laterodorsal thalamic nucleus, dorsomedial part

LDTg laterodorsal tegmental nucleus

LDVL laterodorsal thalamic nucleus, ventrolateral part

Lent lateral entorhinal cortex

lfp longitudinal fasciculus of the pons

LGP lateral globus pallidus

LH lateral hypothalamic area

LHb lateral habenular nucleus

ll lateral lemniscus

LM lateral mammillary nucleus

LMol lacunosum moleculare layer of the hippocampus

lo lateral olfactory tract

LO lateral orbital cortex

LOT nucleus of the lateral olfactory tract

LPAG lateral periaqueductal gray

LPBC lateral parabrachial nucleus, central part

LPBV lateral parabrachial nucleus, ventral part

LPGi lateral paragigantocellular nucleus

LPLR lateral posterior thalamic nucleus, laterorostral part

LPMC lateral posterior thalamic nucleus, mediocaudal part

LPMR lateral posterior thalamic nucleus, mediorostral part

LPO lateral preoptic area

LPtA lateral parietal association cortex

LR4V lateral recess of the 4th ventricle

LRt lateral reticular nucleus

LRtPC lateral reticular nucleus, parvicellular part

LSD lateral septal nucleus, dorsal part

LSI lateral septal nucleus, intermediate part

LSO lateral superior olive

LSS lateral stripe of the striatum

LSV lateral septal nucleus, ventral part

LV lateral ventricle

LVPO lateroventral periolivary nucleus

M1 primary motor cortex

M2 secondary motor sortex

m5 motor root of the trigeminal nerve

MA3 medial accessory oculomotor nucleus

maopt medial accessory optic tract

mcp middle cerebellar peduncle

MCPO magnocellular preoptic nucleus

MD mediodorsal thalamic nucleus

MDC mediodorsal thalamic nucleus, central part

MdD medullary reticular nucleus, dorsal part

MdV medullary reticular nucleus, ventral part

MDL mediodorsal thalamic nucleus, lateral part

MDM mediodorsal thalamic nucleus, medial part

Me medial amygdaloid nucleus

Me5 mesencephalic trigeminal nucleus

me5 mesencephalic trigeminal tract

Med medial cerebellar nucleus

MeDL medial cerebellar nucleus, dorsolateral protuberance

MePD medial amygdaloid nucleus, posterodorsal part

MePV medial amygdaloid nucleus, posteroventral part

mfb medial forebrain bundle

MG medial geniculate nucleus

MGD medial geniculate nucleus, dorsal part

MGM medial geniculate nucleus, medial part

MGP medial globus pallidus

MGV medial geniculate nucleus, ventral part

MHb medial habenular naucleus

Mi mitral cell layer of the olfactory bulb

MiTg microcellular tegmental necleus

ML medial mammillary nucleus, lateral part

ml medial lemniscus

mlf medial longitudinal fasciculus

MM medial mammillary nucleus, medial part

MnA median accessory nucleus of the medulla

MnPO median preoptic nucleus

MnR median raphe nucleus

MO medial orbital cortex

Mol molecular layer of the dentate gyrus

mp mammillary peduncle

MPtA medial parietal association cortex

MPA medial preoptic area

MPB medial parabrachial nucleus

MS medial septal nucleus

MT medial terminal nucleus of the accessory optic tract

mt mammillothalamic tract

mtg mammillotegmental tract

MVeMC medial vestibular nucleus, magnocellular part

MVePC medial vestibular nucleus, parvicellular part

MVPO medioventral periolivary nucleus

MZMG marginal zone of the medial geniculate

ns nigrostriatal bundle

O nucleus O

ocb olivocochlear bundle

ON olfactory nerve layer

Op optic nerve layer of the superior colliculus

opt optic tract

Or oriens layer of the hippocampus

OT nucleus of the optic tract

P5 peritrigeminal zone

P7 perifacial zone

Pa4 paratrochlear nucleus

Pa5 paratrigeminal nucleus

Pa6 paraabducens nucleus

PaAP paraventricular hypothalamic nucleus, anterior parvicellular part

PAG periaqueductal gray

PaLM paraventricular hypothalamic nucleus, lateral magnocellular part

PaV paraventricular hypothalamic nucleus, ventral part

PaS parasubiculum

PBP parabrachial pigmented nucleus

PC paracentral thalamic nucleus

pc posterior commisure

PC5 parvicellular motor trigeminal nucleus

pcn precentral fissure

PCRtA parvicellular reticular nucleus, alpha part

pcuf preculminate fissure

Pe periventricular hypothalamic nucleus

PF parafascicular thalamic nucleus

PFl paraflocculus

pfs parafloccular sulcus

PH posterior hypothalamic area

PIL posterior intralaminar thalamic nucleus

Pir piriform cortex

PL paralemniscal nucleus

PLCo posterolateral cortical amygdaloid nucleus

plf posterolateral fissure

PLi posterior limitans thalamic nucleus

pm principal mammillary tract

PM paramedian lobule

PMCo posteromedial cortical amygdaloid nucleus

PMn paramedian reticular nucleus

PMnR paramedian raphe nucleus

PMV premammillary nucleus, ventral part

Pn pontine nuclei

PnC pontine reticular nucleus, caudal part

PnO pontine reticular nucleus, oral part

Po posterior thalamic nuclear group

PoDG polymorph layer of the dentate gyrus

PoT posterior thalamic nuclear group, triangular part

PP peripeduncular nucleus

ppf prepyramidal fissure

PPT posterior pretectal nucleus

PPTg pedunculopontine tegmental nucleus

PPy parapyramidal nucleus

Pr prepositus nucleus

PR prerubral field

Pr5DM principal sensory trigeminal nucleus, dorsomedial part

Pr5VL principal sensory trigeminal nucleus, ventrolateral part

PrC precommissural nucleus

prf primary fissure

PRh perirhinal cortex

PrL prelimbic cortex

PrS presubiculum

PS parastrial nucleus

psf posterior superior fissure

PSol parasolitary nucleus

PSTh parasubthalamic nucleus

PT paratenial thalamic nucleus

pv periventricular fiber system

PVA paraventricular thalamic nucleus, anterior part

PVP paraventricular thalamic nucleus, posterior part

py pyramidal tract

Py pyramidal cell layer of the hippocampus

pyx pyramidal decussation

Rad stratum radiatum of the hippocampus

RC raphe cap

Re reuniens thalamic nucleus

rf rhinal fissure

Rh rhomboid thalamic nucleus

RLi rostral linear nucleus of the raphe

RMC red nucleus, magnocellular part

RMg raphe magnus nucleus

Ro nucleus of Roller

ROb raphe obscurus necleus

RPa raphe pallidus nucleus

RPC red nucleus, parvicellular part

RPO rostral periolivary region

RRF retrorubral field

rs rubrospinal tract

RSA retrosplenial agranular cortex

RSG retrosplenial granular cortex

Rt reticular thalamic nucleus

RtTg reticulotegmental nucleus of the pons

RVL rostroventrolateral reticular nucleus

S subiculum

S1 primary somatosensory cortex

S1BF primary somatosensory cortex, barrel field

S1DZ primary somatosensory cortex, dysgranular region

S1FL primary somatosensory cortex, forelimb region

S1HL primary somatosensory cortex, hindlimb region

S1J primary somatosensory cortex, jaw region

S1Tr primary somatosensory cortex, trunk region

S1ULp primary somatosensory cortex, upper lip region

S2 secondary somatosensory cortex

Sag sagulum nucleus

SChDM suprachiasmatic nucleus, dorsomedial part

SChVL suprachiasmatic nucleus, ventrolateral part

SCO subcommissural organ

scp superior cerebellar peduncle

sf secondary fissure

SFi septofimbrial nucleus

SFO subfornical organ

SG suprageniculate thalamic nucleus

SGl superficial glial zone of the cochlear nuclei

SHi septohippocampal nucleus

Sim simple lobule

SI substantia innominata

SL semilunar nucleus

SLEAC sublenticular extended amygdala, central part

SLEAM sublenticular extended amygdala, medial part

SLu stratum lucidum, hippocampus

sm stria medullaris of the thalamus

SMT submammillothalamic nucleus

SMV superior medullary velum

SNC substantia nigra, compact part

SNL substantia nigra, lateral part

SNR substantia nigra, reticular part

SO supraoptic nucleus

sol solitary tract

SolC nucleus of the solitary tract, commissural part

SolCe nucleus of the solitary tract, central part

SolDL solitary nucleus, dorsolateral part

SolDM nucleus of the solitary tract, dorsomedial part

SolG nucleus of the solitary tract, gelatinous part

SolI nucleus of the solitary tract, interstitial part

SolIM nucleus of the solitary tract, intermediate part

SolM nucleus of the solitary tract, medial part

SolV solitary nucleus, ventral part

SolVL nucleus of the solitary tract, ventrolateral part

SOR supraoptic nucleus, retrochiasmatic part

sox supraoptic decussation

sp5 spinal trigeminal tract

Sp5C spinal trigeminal nucleus, caudal part

Sp5I spinal trigeminal nucleus, interpolar part

Sph sfenoid nucleus

SPF subparafascicular thalamic nucleus

SPO superior paraolivary nucleus

SpVe spinal vestibular nucleus

st stria terminalis

STh subthalamic nucleus

Su3 supraoculomotor periaqueductal gray

Su3C supraoculomotor cap

Sub submedius thalamic nucleus

SubB subbrachial nucleus

SubG subgeniculate nucleus

SubI subincertal nucleus

SuG superficial gray layer of the superior colliculus

Te terete hypothalamic nucleus

TeA temporal association cortex

ts tectospinal tract

tz trapezoid body

Tz nucleus of the trapezoid body

V1 primary visual cortex

V2L secondary visual cortex, lateral area

V2ML secondary visual cortex, mediolateral area

V2MM secondary visual cortex, mediomedial area

VA ventral anterior thalamic nucleus

VCA ventral cochlear nucleus, anterior part

VDB nucleus of the vertical limb of the diagonal band

VEn ventral endopiriform nucleus

vhc ventral hippocampal commisure

VL ventrolateral thalamic nucleus

VLGMC ventral lateral geniculate nucleus, magnocellular part

VLGPC ventral lateral geniculate nucleus, parvicellular part

VLPAG ventrolateral periaqueductal gray

VM ventromedial thalamic nucleus

VMHC ventromedial hypothalamic nucleus, central part

VMHDM ventromedial hypothalamic nucleus, dorsomedial part

VMHVL ventromedial hypothalamic nucleus, ventrolateral part

VMPO ventromedial preoptic nucleus

VO ventral orbital cortex

VP ventral pallidum

VPL ventral posterolateral thalamic nucleus

VPM ventral posteromedial thalamic nucleus

VRe ventral reuniens thalamic nucleus

vsc ventral spinocerebellar tract

VTA ventral tegmental area

VTg ventral tegmental nucleus

vtgx vetral tegmental decussation

VTM ventral tuberomammillary nucleus

VTRZ visual tegmental relay zone

VTT ventral tenia tecta

TS triangular septal nucleus

Tu olfactory tubercle

X nucleus X

Xi xiphoid thalamic nucleus

xscp decussation of the superior cerebellar peduncle

ZI zona incerta

ZID zona incerta, dorsal part

ZIV zona incerta, ventral part

ZL zona limitans

Zo zonal layer of the superior colliculus
